# Successful reduction of alopecia induced by anthracycline and taxane containing adjuvant chemotherapy in breast cancer – clinical evaluation of sensor-controlled scalp cooling

**DOI:** 10.1186/2193-1801-3-500

**Published:** 2014-09-05

**Authors:** Kay Friedrichs, Martin H Carstensen

**Affiliations:** Mammazentrum Hamburg at Jerusalem Hospital, Moorkamp 2-6, 20357 Hamburg, Germany

**Keywords:** Breast cancer, Adjuvant chemotherapy, Scalp cooling, Alopecia

## Abstract

**Background:**

Scalp cooling is a long known method to reduce chemotherapy-induced alopecia in cancer patients with solid tumors. Due to a progress in this method, a medical device enabling individual feedback-controlled temperature regulation was evaluated.

Between June 2011 and December 2012, 83 breast cancer patients were included. Evaluation was focussed on the quantification of alopecia, satisfaction and side effects of the scalp cooling system in (neo-) adjuvant chemotherapy regimens. Alopecia quantification was done by patient evaluation and experts rating.

**Findings:**

Based on patient hair loss evaluations, the mean overall success rate of scalp cooling (<50% hair loss) in (neo-) adjuvant chemotherapy was at 52.6%. 51.7% of patients in (neo-) adjuvant CT did not need head covers. In 51.7% of patients in (neo-) adjuvant chemotherapy hair regrowth occurred. Patient satisfaction rate was between VAS 70 and 80 (0–100, where 100 is completely satisfied).

**Conclusion:**

The evaluation demonstrates that feedback-controlled scalp cooling provides a good chance for breast cancer patients to keep their hair even during (neo-)adjuvant chemotherapies, which are known to cause severe to complete alopecia without scalp cooling.

## Introduction

Chemotherapy-induced alopecia (CIA) causes considerable impact on the quality of life of patients during cancer treatment. CIA starts approximately within 2–4 weeks and is complete 1–2 months after the initiation of chemotherapy (CT) (Batchelor 2001). Although a number of publications recently refer to the problem of CIA (e.g. (Komen et al. 2013; Van den Hurk et al. 2010)), scalp cooling (SC) as suitable solution is often not offered to patients.

Various SC devices exist since the 1970’s. Their efficacy, handling and control are limited. Particularly a direct temperature control and regulation of the scalp during cooling procedure was not possible. Although several publications report the efficacy of modern systems, SC still has the image of being ineffective (Tollenaar et al. 1994; Middleton et al. 1985).

The DigniCap® System (Dignitana, Sweden) is a new medical device for SC treatment during CT. The system enables feedback-controlled regulation of SC by direct measurement of the patient’s individual scalp temperature.

The clinical evaluation of the system was performed at Mammazentrum Hamburg. This evaluation was focussed on the results for hair loss, patient satisfaction and SC related side effects in breast cancer patients treated with (neo-)adjuvant CTs.

## Material and methods

The evaluated SC system consists of a computer controlled refrigerator unit connected through hoses to a silicon cap in which liquid coolant circulates through small channels. Scalp temperature is monitored by two sensors in the silicone cap independently controlling scalp temperature of the front and the back side of the head, plus one safety sensor to ensure that the temperature does not go below the freezing point.

The SC procedure starts at room temperature with the fit of the silicone cap on the patients head. Patient hair is wetted to ensure better heat conductivity. The silicone cap is covered by an insulating neoprene cap. If follows the pre-cooling time of usually 20 minutes were the patients scalp is continually cooled down to the pre-set treatment temperature (3-5°C). This temperature is sensor-controlled and kept during the following CT infusion time. After the CT infusion is finished, the SC is continued for the post-cooling time. The post-cooling time protects the hair follicles from the peak toxicity of the CT drugs. The duration of the post cooling time depends on the CT regimen (30–150 minutes).

Between June 2011 and December 2012 a total of 83 breast cancer patients were included in the evaluation. Inclusion criteria were: Age ≥18 years; female; signed informed consent; confirmed primary or metastasized breast cancer; normal hair distribution at baseline as judged by a physician; ECOG performance status 0–2; assigned for CT; capacity and willingness to comply with study procedure; estimated life expectance must be greater than 6 months. Exclusion criteria were: prior administration of chemotherapy within 2 years of recruitment; prior radiotherapy treatment involving the head; inherent alopecia due to other causes; any conditions which have contraindications mentioned in the IFU of the evaluated system.

Table 1 shows the types of applied CTs, the corresponding number of patients who finished CT and SC and the CT-specific post-cooling times. Selected characteristics (age, menopausal status and ethnic phenotype) of the patient cohort are summarized in Table 2. 58 patients received a (neo-)adjuvant chemotherapy with SC, six patients were treated in a palliative setting. As the number of patients in palliative treatment is relatively low the results are not separated by CT regimen.

**Table 1:** **Chemotherapy regimens, dosages, post-cooling times and number of patients who finished the DigniCap evaluation**

Chemotherapy regimen and doses	No of patients	Post-cooling time (min)
**(Neo-)adjuvant CT**		
E_90_C_600_ (q3w*4) - > Doc_100/175_ (q3w*4)^1^	32	100
F_500_E_100_C_500_ (q3w*6)	15	120
F_500_E_100_C_500_ (q3w*3) - > Doc_100_ (q3w*3)	7	120 - > 100
E_90_C_600_ (q3w*4)	3	100
CarboPt/Doc (q3w*6)	1	90
**Palliative CT**		
Taxol_135_/Herceptin_8mg/kg_	1	90
Halaven_1.23_	1	120
Taxol_90_/Avastin_10mg/kg_	3	60
Carboplatin	1	90
Gemcitabine/Cisplatin	(1^2^)	60

^1^Three patients were included receiving three-weekly (neo-)adjuvant Doc/EC. Of them, one patient was treated with Doc_175_ and two with Doc_100_.

^2^One patient finished an adjuvant CT (EC/Doc) before a palliative treatment (three cycles Carboplatin followed by two cycles Gemcitabine/Cisplatin) was started.

**Table 2:** **Selected characteristics of the patient cohort in the clinical evaluation**

Median age	
(neo-)adjuvant CT scalp cooling finished n = 58	47 years (range: 28–74 years)
Nalliative CT scalp cooling finished n = 6	47 years (range: 25–64 years)
**Menopausal status**	
(neo-)adjuvant CT	premenopausal: 37 patients
	postmenopausal: 32 patients
Palliative CT	premenopausal: 1 patient
	postmenopausal: 5 patients
No information	8 patients
**Ethnic phenotype**	
Caucasian	81 patients
Asian	1 patient (neo-adjuvant CT)
African	1 patient (neo-adjuvant CT)

Anonymized patient data were gathered by completion of questionnaires after each CT-cycle. Patients evaluated their hair loss, their satisfaction and SC-related magnitude of different possible side effects on a visual analog scale (VAS, range 0–100) in the following matter:

No hair loss - total hair loss; completely unsatisfied - completely satisfied; no side effect – strongest possible side effect.

Moreover patients were asked whether and when they used a head cover. Every patient who wore a head cover at least once during CT, was rated as head cover user.

A set of photographs was taken from each patient before each CT-cycle. After the patients faces were covered and records were anonymized, the sets of photographs were randomized for CT-cycle and rated for hair loss and hair regrowth by an expert from the Institute of Cosmetic Science, Hamburg University, Dept. of Chemistry, Division of Biochemistry and Molecular Biology to provide an objective rating. Rating categories were defined according to a modified version of Dean’s scale for hair loss (Table 3). Scientific literature offers several different scales to evaluate the extent of hair loss during CT. Many authors refer to the WHO scale for hair loss (World Health Organisation 1979; Massey 2004) although modifications are often not indicated. As the WHO scale has the disadvantage that hair loss cannot be expressed quantitatively, which hampers statistical analysis, some authors prefer Dean’s scale for hair loss quantification (Dean et al. 1979). This scale with original four percentage-based categories of hair loss was modified to the five categories of the WHO scale (e.g. Rugo et al. 2013). However, Dean’s scale categories should not be directly compared with grading criteria scales that base on WHO (Connie et al. 2004). Table 3 shows a comparison of hair loss scales used in scientific publications. The hair loss scale used in this evaluation (Table 3) is a modified version of Dean’s scale (Rugo et al. 2013), slightly changed to avoid the value overlap bias of the original scale.

**Table 3:** **Selected different classifications for hair loss evaluation found in the literature**

	Grade 0	Grade 1	Grade2	Grade 3	Grade 4
WHO (1979)	No change	Minimal hair loss	Moderate, patchy alopecia	Complete alopecia, but reversible	Complete and non-reversible alopecia
Massey (2004)	No significant hair loss	Minor hair loss	Moderate hair loss	Significant hair loss	Total hair loss
Dean (1979)	0-25%	25-50%	50-75%	75-100%	-
Rugo (2013)	0%	<25%	25-50%	50-75%	>75%
This evaluation	0%	1-24%	25-49%	50-74%	75-100%

According to Rugo et al. (2013) scalp cooling is successful with a hair loss ≤50% (Grades 0, 1 and 2) as 50% is the threshold where hair loss becomes noticeably visible (Breed et al. 2011). Based on this categorization, the success rate of SC in this evaluation was calculated at the end of the CTs (patient evaluation/experts rating).

To enable comparability between expert rating and patients’ evaluations, patients VAS hair loss evaluations were transferred to percentages and grouped in the Dean’s scale for hair loss before data analysis.

Statistical analysis of the data was performed by medistat GmbH, Kiel, Germany. Focus was set on the efficacy of the SC system manifested in the rate of hair loss, number of patients using head cover, SC-related side effects, regrowth of hair and the satisfaction of the patients with the amount of preserved hair.

## Results

### Scalp cooling during (neo-)adjuvant chemotherapy

The overall success rate of SC during (neo-)adjuvant CT calculated from patient evaluation and experts rating (PE and ER) is 52.6%/53.5% (Table 4). The overall success rate in the group with the most frequently applied CT-regime (EC/Doc) is 46.8%/53.5%. Due to the low number of cases in other CT-regimens, the number of patients with successful SC might be more informative than percentages. Patients treated with EC (3 patients): 2 (PE)/1 (ER); patients treated with FEC (17 patients): 9 (PE)/9 (ER); patients treated with FEC/Doc (7 patients): 4 (PE)/2 (ER).

**Table 4:** **Mean hair loss at the end of CT (last rated CT-cycle) evaluated by patients (PE) and rated by expert (ER) Threshold for successful scalp cooling is 50% (visible) hair loss, i.e. the limit between grades 2 and 3 (n (%))**

Grade (hair loss)	Success			No success	
	0	1	2	3	4
**(Neo-)adjuvant CT**					
**Success rate**	31 (52.6)			28 (47.5)	
**Σ all CT** PE					
**Σ all CT** ER	31 (53.5)			27^1^ (46.6)	
PE	1 (1.7)	7 (11.9)	23 (39.0)	19 (32.2)	9 (15.3)
ER^1^	3 (5.2)	12 (20.7)	16 (27.6)	19 (32.8)	8 (13.8)
**Success rate**	2 (66.6)			1 (33.3)	
**Σ all EC** PE					
**Σ all EC** ER	1 (33.3)			2 (66.6)	
**EC** PE	-	1 (33.3)	1 (33.3)	-	1 (33.3)
**EC** ER	-	1 (33.3)	-	1 (33.3)	1 (33.3)
**Success rate**	15 (46.8)			17 (53.1)	
**Σ all EC/Doc** PE					
**Σ all EC/Doc** ER	17 (54.9)			14 (45.2)	
**EC/Doc** PE	1 (3.1)	5 (15.6)	9 (28.1)	13 (40.6)	4 (12.5)
**EC/Doc** ER^1^	2 (6.5)	6 (19.4)	9 (29.0)	11 (35.5)	3 (9.7)
**Success rate**	9 (60.0)			6 (40.0)	
**Σ all FEC** PE					
**Σ all FEC** ER	9 (60.0)			6 (40.0)	
**FEC** PE	-	1 (6.7)	8 (53.3)	3 (20.0)	3 (20.0)
**FEC** ER	1 (6.7)	5 (33.3)	3 (20.0)	5 (33.3)	1 (6.7)
**Success rate**	4 (57.1)			3 (42.9)	
**Σ all FEC/Doc** PE					
**Σ all FEC/Doc** ER	2 (28.6)			5 (71.5)	
**FEC/Doc** PE	-	-	4 (57.1)	2 (28.6)	1 (14.3)
**FEC/Doc** ER	-	-	2 (28.6)	2 (28.6)	3 (42.9)
**Palliative CT**					
**Success rate**	6 (100)			-	
**Σ all** PE					
**Σ all** ER	5 (83.4)			1 (16.7)	
PE	1 (16.7)	4 (66.7)	1 (16.7)	-	-
ER	3 (50)	1 (16.7)	1 (16.7)	1 (16.7)	-

^1^The African patient was not rated.

28/58 patients (48.3%) receiving (neo-)adjuvant CT used head covers (Table 5) like wig, cap or scarf. 17/29 patients (58.6%) used their head cover permanently, whereas 12/29 patients (41.4%) used a head cover sometimes, e.g. when going out.

**Table 5:** **Use of head cover by chemotherapy (n/%)**

Use of head cover	always	sometimes
**Total**	17/26.6	12/18.8
**(Neo-) adjuvant CT**		
all	16/27.6	12/20.7
EC	1	-
EC/Doc	9	10
FEC	3	-
FEC/Doc	3	2
**Palliative CT**	**1**	**-**

**Figure 1:**
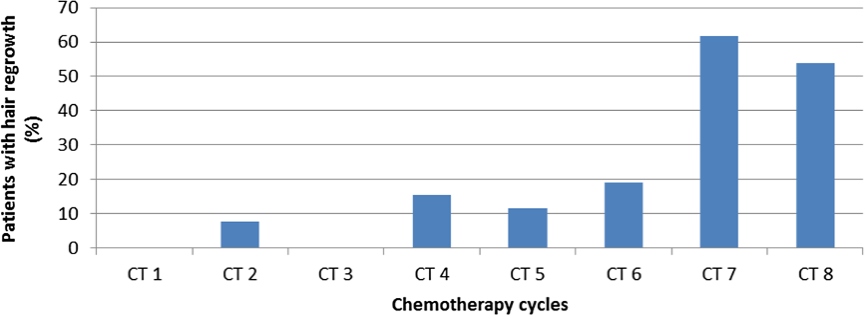
**Percentages of patients with hair regrowth (n = 20) in breast cancer patients under EC/Doc (n = 26).** The observed regrowth is shown due to specific CT-cycle.

Hair regrowth was observed on photographs in 30/58 (51.7%) patients receiving a (neo-)adjuvant CT. Major contribution came from 26 patients treated with EC/Doc where 20 patients were rated with hair regrowth (76.9%). Figure 1 shows that in this subgroup hair regrowth mainly occurred in the second half of CT. In all three patients receiving Doc/EC included in the analysis of this group, hair regrowth was observed under EC, i.e. in the second part of the CT. All visible hair regrowth was made up by regular hair and not by the often observed fine and curly chemotherapy-hair (Katakunnel & Berger 2008).

Satisfaction evaluation with respect to hair preservation and the scalp cooling treatment (Table 6) provided comparable results with (mean/median) VAS 71/75 and VAS 73/80 satisfaction of all patients receiving (neo-)adjuvant CT.

**Table 6:** **Satisfaction evaluation for hair preservation at the end of CT (last rated CT-cycle) and with the scalp cooling treatment in VAS (0–100)**

Satisfaction with hair preservation					
(Neo-) adjuvant CT	mean	sd	median	max	min
all	71	25	75	100	8
EC	70	20	70	90	50
EC/Doc	71	28	80	100	8
FEC	75	20	75	100	30
FEC/Doc	57	24	60	85	15
**Palliative CT**	**92**	**11**	**95**	**100**	**70**
**Satisfaction with scalp cooling treatment**					
**(Neo-) adjuvant CT**					
all	73	26	80	100	1
EC	68	18	65	90	50
EC/Doc	71	28	80	100	1
FEC	82	18	80	100	20
FEC/Doc	65	25	70	100	10
**Palliative CT**	**94**	**10**	**100**	**100**	**50**

### Scalp cooling during palliative chemotherapy

All six patients evaluated SC as successful receiving palliative CT’s. Five patients were rated as success by the expert (Table 4).

One patient treated with palliative chemotherapy used a head cover (Table 5).

Patients treated with palliative CT were generally more satisfied (Table 6) than patients in (neo-)adjuvant CT with (mean/median) VAS 92/95 (hair preservation) and VAS 94/100 (scalp cooling treatment).

### All patients

Side effects of scalp cooling were evaluated by the patients on a VAS scale. Table 7 summarizes the side effects most frequently rated as strong, i.e. ≥50%. Only 2 out of 19 patients who stopped the SC treatment, stopped due to physical side effects (one due to feeling cold and one due to headache). The frequency of side effects was generally higher in patients with (neo-)adjuvant CT compared to patients in palliative CT regimens. Feeling cold was the most frequent side effect in (neo-)adjuvant treatments (37.9%), followed by headache (24.1%), heaviness of head (18.9%) and scalp pain (10.3%). Patients with palliative treatment reported feeling cold in two cases (33.3%) and scalp pain in one case (16.7%) as the most frequent side effects.

**Table 7:** **The most important side effects rated as strong by patients (VAS ≥50)**

Side effect ≥50%	Patients with adjuvant CT (n = 58)	Patients with palliative CT (n = 6)
Feeling cold	22 (37.9%)	2 (33,3%)
Headache	14 (24.1%)	0
Heaviness of head	11 (18.9%)	0
Scalp pain	6 (10.3%)	1 (16,7%)

64/83 (77%) patients finished CT and SC treatment (Table 1). Of the 19 patients who dropped-out, nine patients (10.8%) stopped due to unspecified intolerance, five (6.0%) patients due to hair loss, three patients (3.6%) due to cancer related emergency cases or disease progression and two patients (2.4%) due to SC-related side effects (feeling cold, headache).

## Discussion

The success of scalp cooling and other related results (e.g. patient satisfaction) is very much dependent on the applied CT-regimens (Komen et al. 2013). Different methods for the prevention of CIA are used since the 1970ies. For some SC methods, high discomfort and weak success rates needed to be tolerated by the patients (Breed et al. 2011). This might be the main reasons why SC is still not broadly accepted. However, since CIA is one of the most distressing side effects of CT, several efforts for improvement of SC are done. Some crucial factors for successful SC are meanwhile identified, e.g.:

1. Optimal fit of the cooling cap, target temperature, temperature control, device handling and logistics.
2. The type of drugs and their combinations and the individual drug doses.

One technique for SC is based on gel caps. This requires multiple caps that need to be changed several times during treatment and a refrigerator unit for cooling. The cooling has no temperature control and the optimal fit of the caps needs to be found after each change of the cap and therefore causes high discomfort for the patients. The more advanced technology of liquid cooled caps still has the drawback of lacking temperature control. Only the system in this evaluation provides a feedback-controlled temperature regulation independent for the backside of the head and for the forehead, ensuring optimal SC temperature for every individual patient at any time.

Strong alopecia is particularly caused by anthracyclines (e.g. epirubicin, adriamycin) (Breed et al. 2011). Even stronger toxicity towards the hair roots is expected for combinations of anthracyclines with taxanes, which are belonging to the most frequently applied regiment for treatment of primary breast cancer. Since a variety of (neo-)adjuvant CT regimens are used in different dosages, mixed with different contributions of palliative CT-regimens, all these factors need to be taken into account when comparing study results. In our study 54/58 patients were treated according to the guidelines of AGO breast commitee (AGO e.V) with a combination of anthracycline (dose of 90–100 mg/m^2^) and taxane (dose ≥100 mg/m^2^). In all cases, epirubicin was combined with cyclophosphamide (500 or 600 mg/m2). Mols et al. (2009) found for different adjuvant CT-regimens, that breast cancer patients who did not receive SC reported average hair loss VAS 85 (range 9 to 100), where 100 is complete hair loss. This supports the common experience that current (neo-)adjuvant CT for breast cancer is causing complete alopecia in almost all cases.

In our study we considered the evaluation and rating of hair loss at the final CT-cycle as the most relevant result of scalp cooling. The mean success rate of SC in patients under (neo-)adjuvant CT (patient evaluation/experts rating) was 52.6%/53.5% and for patients in palliative treatment (patient evaluation/experts rating) 100%/83.3%. Remarkable are the very comparable results of patient evaluation and expert rating. This suggests that, although under the impression of the distressing impact of chemotherapy, patients were able to neutrally quantify their hair loss. In the entire Dutch Scalp Cooling Registry, with a 71% contribution of several adjuvant and even including 29% of different palliative CT regimens known to cause less CIA, a success rate of 50% was found based on a non-use of wigs (Van den Hurk et al. 2012). Breed at al. (2011) published SC results in The Netherlands between 2006 and 2009 separated by CT regimens. They found a success rate for FEC (drug doses: 500/100/500 mg/m^2^) and FEC- > Doc (drug doses: 500/100/500- > 100 mg/m^2^) SC success rates of 47% and 33%. In our study the success rate of feedback controlled SC is higher with (PE/ER) 60.0%/60.0% and 57.1/28.6% in FEC and FEC/Doc CT regimens.

Palliative CT regimens generally cause less alopecia (Van den Hurk et al. 2012), which is also supported by our data as all patients treated with palliative CT in this study evaluated their scalp cooling as success. Even the patient who was rated by the expert to no success (Table 4) filed a satisfied evaluation (VAS 90).

In some publications the SC success rate was indirectly calculated from head cover use (Van den Hurk et al. 2010; Mols et al. 2009). According to our evaluation of head cover use, 51.7% of the patients did not use any head cover, although all of them received (neo-)adjuvant CT-regimens in comparatively high doses.

The frequency of wearing a head cover might be influenced by culture, self-esteem, sex and mentality of the patients. Moreover, like the success rate of SC, the head cover rate is very much dependent on CT regime and its dosage. For example by increasing the epirubicin dose in the FEC-regime from 90 to 100 mg/m^2^, the rate of patients using a head cover increased from 33% to 52%, respectively (Katakunnel & Berger 2008). Van den Hurk et al. (2010) and Mols et al. (2009) showed that 52% of breast cancer patients in various adjuvant CTs with SC did not use a wig while only 2.7% of the control group required none. In another study van den Hurk et al. (2012) found a head cover rate of 49% with a change from 5% (no cooling) to 49% (cooling, factor 10).

Within the group of patients receiving (neo-)adjuvant CTs, the rate of hair regrowth in our study was at 51.7%. It was also observed that hair regrowth mostly started in the second half of CT, e.g. under Doc in the EC/Doc regime). As hair regrowth could be observed when EC was applied in the second half of CT, this suggests that regrowth might be not only influenced by drug type, but also by the time period since initiation of alopecia. Moreover, even in cases where SC was not able to completely prevent alopecia, the observation of hair regrowth a certain time after initial hair loss suggests that SC has at least some protective effect on the hair follicles, since hair regrowth occurred relatively early and with normal hair quality and density.

Based on this assumption, we hypothesise that SC might also have a prophylactic effect in prevention of permanent CIA, which is known to occur in CT’s containing cyclophosphamide, carboplatin, doxetacel and paclitaxel (Breed et al. 2011; Prevezas et al. 2009). However, this needs further investigation. Reports about hair regrowth during CT are rare. Most relevant publications provide regrowth data a couple of weeks or even months after CT. Van den Hurk et al. (2010) found lower rates of hair regrowth during CT of 24% studying a patients cohort with solid tumors (mainly breast cancer) treated with different adjuvant and palliative CT-regimens. The control group of the study (no SC) had a rate of hair regrowth of 7%.

Our satisfaction evaluation was focussed on hair preservation and the SC treatment just after the last CT-cycle. In summary, an average satisfaction rate between VAS 70 and 80 was found. Van den Hurk et al. (2013) provided data about CIA on a VAS scale and the satisfaction with the patients’ hair style 3 weeks after finishing CT. They found 85% satisfaction (SC patients) and 78% satisfaction in the control group (without SC). The high degree of patient satisfaction also in the control group suggests that patient satisfaction at least in this study is not strongly dependent on CIA. Studies to further investigate the reasons determining patient satisfaction are necessary.

SC was generally well tolerated as only two dropouts (2.4%) were due to SC-specific physical side effects. 9/83 (10.8%) patients stopped due to unspecific intolerance, which at least in some cases might be summarized as side effects anticipated from chemotherapy. In total 77.1% of breast cancer patients included in this study finished the treatment. Patients were treated with heating blankets, hot beverages or in some cases with pain killers, e.g. Ibuprofen 400. The four most prominent SC related side effects in this evaluation (Table 7) were also the most frequent in several other studies (Breed et al. 2011). However, due to the feedback-controlled temperature regulation, the evaluated SC system provides results among the best tolerance scores published in literature. Based on several studies, the mean tolerance of SC was calculated to VAS 6.9 to 8.0 (range 0–10) with 10 as “really well tolerable” (Breed et al. 2011).

## Conclusions

This evaluation of the DigniCap System demonstrated that feedback-controlled SC provides a good chance for breast cancer patients to keep their hair during combined anthracycline and taxane containing (neo-)adjuvant CTs. Even under initial hair loss, there is a reasonable chance to get hair regrowth during continued SC and CT. SC-related side effects are mild, the cooling treatment is well tolerated and possibly most important, provides a high degree of patient satisfaction.

### Ethical standards

The conducted experiments comply with the current laws of Germany. This evaluation was approved by the related ethics vote File no. A144/10, approved by the ethics committee of the Faculty for Medicine, University Kiel, Germany).
